# Penpulimab in an adolescent patient with pancreatic neuroendocrine carcinoma with liver metastasis: A case report and literature review

**DOI:** 10.1097/MD.0000000000042730

**Published:** 2025-06-06

**Authors:** Yafen Li, Jing Zhang, Jie Zheng, Yujie Liu, Lu Zhao, Zhiyu Ma

**Affiliations:** a Department of Clinical Pharmacy, Wanbei Coal and Electricity Group General Hospital, Suzhou, Anhui, China; b General Clinical Research Center, Wanbei Coal and Electricity Group General Hospital, Suzhou, Anhui, China; c Department of Radiotherapy, Wanbei Coal and Electricity Group General Hospital, Suzhou, Anhui, China; d Department of Oncology, Wanbei Coal and Electricity Group General Hospital, Suzhou, Anhui, China.

**Keywords:** anlotinib, case report, immunotherapy, pancreatic neuroendocrine carcinoma, penpulimab

## Abstract

**Rationale:**

Pancreatic neuroendocrine carcinoma (PNEC) is a rare, aggressive malignancy with limited therapeutic options and a dismal prognosis, particularly in young patients. Approximately 85% of PNEC cases eventually progress to metastatic cancer. Despite advances in oncology, optimal management strategies for metastatic PNEC remain undefined, especially in chemotherapy-ineligible cases.

**Patient concerns:**

An 18-year-old male patient, reporting anorexia with associated weight loss (15 kg weight loss over 3 months) and significant abdominal distension.

**Diagnoses:**

The results of whole abdominal computed tomography, histopathology, immunohistochemistry, and laboratory examination were consistent with pancreatic neuroendocrine carcinoma with liver metastasis.

**Interventions:**

Due to the family refusal of chemotherapy, and immunohistochemistry revealed programmed death-1 ligand positivity. The patient received penpulimab (anti-programmed death-1) combined with anlotinib capsules and then changed to penpulimab combined with sorafenib capsules after progression.

**Outcomes:**

Initial therapy achieved 13 months of progression-free survival, demonstrating durable disease control. Subsequent progression highlighted challenges of acquired resistance, with no severe treatment-related toxicity. The patient was still alive at the time of follow-up in July 2024.

**Lessons:**

Pancreatic neuroendocrine cancer is rare. There are various treatment options available. However, the best treatment plan still needs further exploration. This case underscores that programmed death-1 ligand + PNEC may respond to immunotherapy/antiangiogenic combinations, offering alternatives for chemotherapy-ineligible patients. In addition, young patients with aggressive PNEC represent an understudied population, necessitating tailored strategies.

## 1. Introduction

Neuroendocrine neoplasms (NENs) are a rare group of tumors that originate from peptidergic neurons and neuroendocrine cells, characterized by neuroendocrine differentiation and the expression of neuroendocrine markers, which can occur in various parts of the body, including the pancreas^[1]^ neuroendocrine tumor/carcinoma (PNET/C) is a particularly rare tumor among adolescent patients. The annual incidence is <1 case per 100,000 individuals.^[2]^ Most PNET/Cs are nonfunctional tumors that are asymptomatic in the early stages and are often diagnosed at an advanced stage. The only cure for both functional and nonfunctional PNET/C is surgical resection.^[3]^ Unfortunately, 85% of pancreatic neuroendocrine carcinoma (PNEC) eventually develop distant metastases, leading to metastatic disease.^[4]^ For advanced or metastatic PNEC, other treatments, such as immunotherapy, biologic therapy, or targeted therapy, should be considered.

Considering the abundance of possible origins, locations, and tumor specifications, there is still no consensus on the optimal treatment regimen for PNEC. As immune checkpoint inhibitors (ICIs) have revolutionized cancer treatment,^[5]^ these include cytotoxic T-lymphocyte antigen 4,^[6]^ programmed death-1 (PD-1), and programmed death-1 ligand (PD-L1),^[7]^ which block the inhibitory effects of tumor cells on immune cells and enhance the immune response, showing significant efficacy in many solid tumors. In addition, the gastroenteropancreatic–NENs (GEP–NENs) tumor microenvironment is enriched with immune cells, including T cells, natural killer cells, among others. Therefore, a variety of ICIs therapeutic approaches are being investigated to stimulate the immune system to inhibit the growth and progression of GEP–NENs.^[8]^ However, the application and effects of ICIs in pancreatic neuroendocrine tumors are still in the research stage, and current data are relatively limited.^[9]^

ICIs have been understudied in PNEC, and most research has focused on GEP–NENs. In GEP–NENs, immunohistochemical staining for PD-L1 has shown expression in approximately 20% of cases.^[10]^ Compared with low-grade neuroendocrine tumors, there is an increased expression of PD-L1 in high-grade (G3) and poorly differentiated neuroendocrine carcinomas, which correlates with a poorer prognosis,^[11]^ PD-1/PD-L1 immunotherapy may be efficacious in high-grade GEP–NENs. In a study assessing PD-L1 status in tumor samples, 21% of pancreatic neuroendocrine tumors were positive for PD-L1 expression, and treatment with pembrolizumab resulted in an objective response in 6% of patients with PNET (95% confidence interval [CI] = 0%–30%). Approximately 88% of patients with PNETs had stable disease (SD).^[12]^ In addition, case reports showed that 2 patients were treated with pembrolizumab or nivolumab monotherapy; 1 patient maintained SD after 16 months of treatment, and the other patient remained stable at the 6-month follow-up. Both patients tolerated the treatment well.^[13]^

Penpulimab (Anico, Annike, Lianyungang, China) was approved by the National Medical Products Administration in August 2021. It functions primarily by binding to the PD-1 receptor, blocking the interaction between PD-1 and PD-L1/PD-L2, thereby inhibiting immune escape by tumor cells and exerting an antitumor effect.^[14]^ Anlotinib is a novel multitarget tyrosine kinase inhibitor used to inhibit VEGFR2/3, FGFR1–4, and PDGFRα/β, among others. The combination of these 2 agents has shown promising results in pancreatic cancer treatment.^[15,16]^ This article reports a case of an adolescent patient with PNEC and multiple metastases, achieving a favorable clinical outcome with combined treatment of penpulimab and anlotinib, which provides a valuable reference for clinical practice.

## 2. Case report

**Figure 1.**
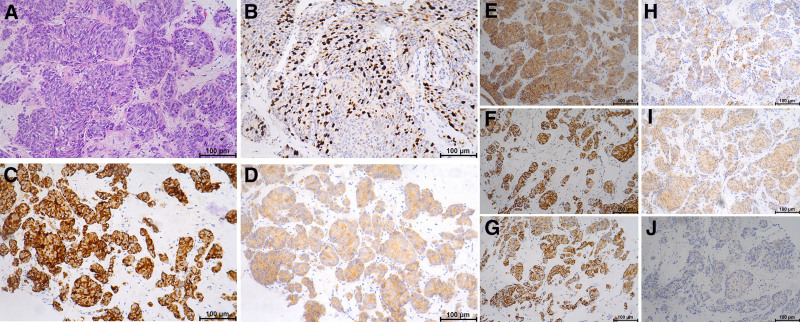
Pathological and immunohistochemical findings of liver biopsies. (A) HE staining showed hepatocyte morphological changes (×20), IHC staining showed that liver cells were positive for (B) Ki-67 (>20%), (C) CgA, (D) Syn, (E) CD56, (F) CK20, (G) CK19, (H) CK7, (I) S100 and negative for (J) HepPar-1, supporting the diagnosis (A–J). CA = carbohydrate antigen, HE = hematoxylin and eosin, IHC = immunohistochemistry.

An 18-year-old male patient, student in college, presented to our hospital on December 24, 2022, reporting anorexia with associated weight loss since September 2022 (15 kg weight loss over 3 months) and significant abdominal distension since December 2022, without other significant symptoms. He reported no family history of malignant tumors. The physical examination revealed moderate nutrition, normal development; superficial lymph nodes were nonpalpable. Abdominal examination revealed distension and tenderness, although the patient was noncooperation examination during the examination. Levels of serum tumor markers were as follows: normal levels included carbohydrate antigen (CA) 19–9 at 13.8 U/mL, CA242 at 4.4 U/mL, CA724 at 2.49 U/mL, CA50 at 23.9 U/mL, and alpha-fetoprotein, 2.40 IU/mL. Abnormal findings included elevated carcinoembryonic antigen at 26.09 ng/mL, total prostate-specific antigen at 49.89 ng/mL, and free prostate-specific antigen at 45.61 ng/mL. Abnormal liver function included aspartate aminotransferase at 129 U/L, mitochondrial isoenzymes of millet grass at 29 U/L, alkaline phosphatase at 179 U/L, and glutamyl aminotrans peptidase at 331 U/L. Routine blood and urine analyses showed no abnormalities. An upper abdominal computed tomography (CT) scan revealed a hypoenhanced region in the pancreatic area, suggesting a possible pancreatic neoplasm. The liver was enlarged with multiple areas of abnormal internal enhancement. On February 3, 2023, a whole abdomen CT examination revealed: multiple diffuse lesions in the pancreatic tail and liver, suggesting a malignant tumor of the pancreatic tail with multiple liver metastases; ascites and a nodule in the left adrenal gland; multiple enlarged lymph nodes in the right inguinal and pelvic wall regions, considered to be metastatic. A percutaneous liver biopsy was performed on February 18, 2023, indicated poorly differentiated carcinoma. Immunohistochemistry results showed: Ki-67 (>20%), CgA (+), Syn (+), CD56 (+), CK20 (+), CK19 (+), CK7 (+), S100 (+), HepPar-1 (−), MUC5AC (−) (Fig. 1), and the expression of PD-L1 was positive.

**Figure 2.**
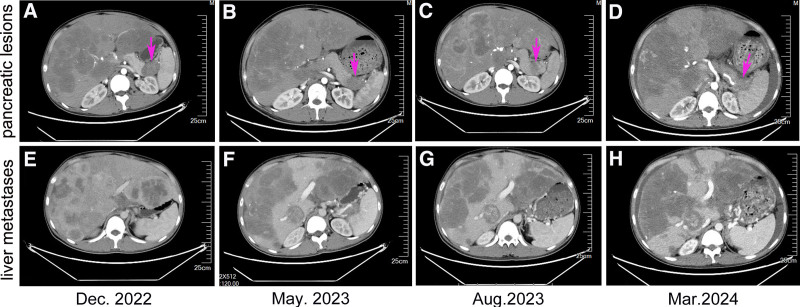
Enhanced CT imaging changes of pancreatic and liver during drug therapy. (A and E) Pancreatic and liver images evaluation before treatment: progressive disease (pink arrows: 4.1 cm in maximum diameter). (B and F) Pancreatic and liver images evaluation after treatment with penpulimab and anlotinib for 4 cycles: stable disease (pink arrows: 3.4 cm in maximum diameter). (C and G) Pancreatic and liver images evaluation after treatment of penpulimab combined with anlotinib for 8 cycles: stable disease (pink arrows: 2.3 cm in maximum diameter). (D and H) Pancreatic and liver images evaluation in March 2024, the lesions were enlarged and increased: progressive disease (pink arrows: 2.9 cm in maximum diameter). CT = computed tomography.

**Figure 3.**
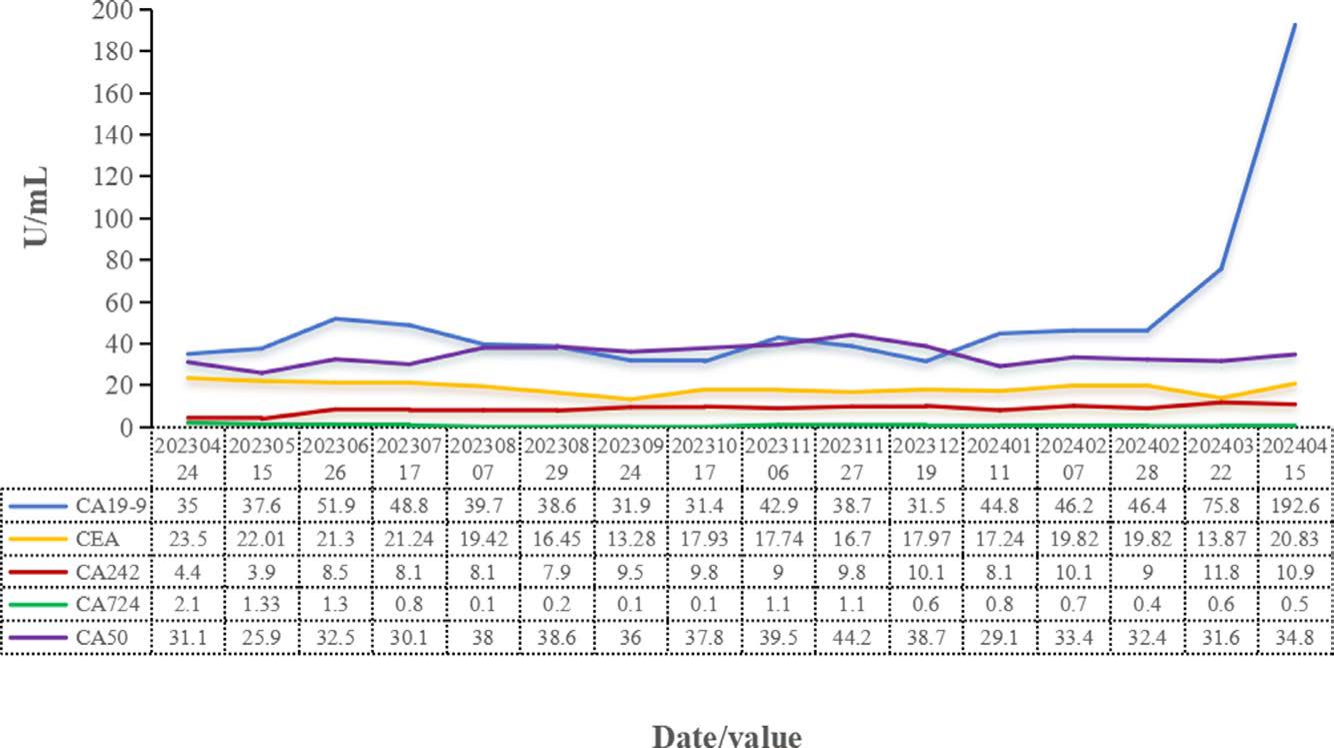
Changes of CA19-9, CEA CA242, CA724, and CA50 during treatment. CA = carbohydrate antigen, CEA = carcinoembryonic antigen.

On the basis of the pathological examination, immunohistochemical results, and detailed clinical history, the patient was diagnosed with liver metastatic PNEC. The patient’s family refused to use chemotherapy drugs, so we considered giving targeted therapy combined with immunotherapy. After obtaining the consent of the patient and their family, the patient began treatment with penpulimab injection (200 mg), administered intravenously once every 3 weeks for immunotherapy, in combination with oral anlotinib capsules (8 mg, q.d.) for targeted therapy from February 20, 2023, along with tramadol sustained-release tablets for pain relief. After 4 cycles of treatment, clinical symptoms were alleviated, with no significant increase in pancreatic and liver tumor lesions, the maximum diameter of pancreatic lesions was 3.4 cm (Fig. 2B and F); the CA19-9 level was rechecked at 37.6 U/mL, indicating SD. This status was maintained until March 22, 2024, when the patient again experienced significant abdominal distension, and the CT scan showed a marked increase and multiplication of liver metastatic lesions, the maximum diameter of pancreatic lesions increased from 2.3 cm to 2.9 mm (Fig. 2D and H), with the CA19-9 level increased from 46.4 to 75.8 U/mL, indicating disease progression. We considered possible anlotinib resistance, on March 26, 2024, the treatment plan was changed to surufatinib capsules, at a dosage of 200 mg orally once daily, in combination with penpulimab (200 mg, intravenous infusion, q3w). On April 15, 2024, the patient was readmitted due to abdominal bloating and abdominal pain, during hospitalization, oxycodone sustained-release tablets were used for pain management, abdominal infusion of ya dan zi oil was administered for auxiliary antitumor treatment, and ascites were drained, the ascites was considered to be transudate by routine and biochemical tests. Local intraperitoneal antitumor therapy was not performed. During the treatment period, the patient experienced grade 1 drug-induced liver injury, and the level of thyroid-stimulating hormone was slightly increased (NCI CTCAE 5.1), which was managed with liver-protective medication and regular monitoring of thyroid-stimulating hormone levels. No other serious adverse reactions were reported. Changes in abdominal-enhanced CT imaging are shown in Figure 2, and changes in tumor markers are shown in Figure 3.

After achieving clinical stabilization, the patient’s family requested discharge on May 1, 2024. The patient subsequently transitioned to outpatient care, with no further diagnostic evaluations conducted during follow-up.

At the time of manuscript preparation in July 2024, the patient was not doing well but was alive. During the combined treatment with penpulimab, the patient’s progression-free survival (PFS) was 13 months.

## 3. Discussion

Pancreatic cancer is a leading cause of cancer-related mortality worldwide, with its incidence increasing annually. It is projected to become the world’s second leading cause of cancer-related mortality by 2030,^[17]^ PNET/Cs account for 1% to 2% of all pancreatic tumors and are considered rare, heterogeneous neoplasms that are believed to originate from embryonic endodermal cells, with an estimated incidence of 1 to 1.5 cases per 100,000 population.^[18]^ Although PNET/Cs have a relatively good prognosis compared with other pancreatic cancers due to their less aggressive nature,^[19]^ they are often not detected due to the nonspecific and intermittent symptoms. Approximately 75% of patients with PNETs present with metastatic disease at diagnosis^[20]^ resulting in a poor prognosis. PNECs most commonly metastasize to the liver, with less frequent metastasis to bone.^[21]^

Treatment of PNET/Cs includes surgical resection, pharmacotherapy, interventional therapy, and peptide receptor radionuclide therapy. Surgical resection remains the primary method.^[22]^ However, >75% of patients with liver metastases have a poor prognosis; therefore, alternative therapies must be considered when surgery is not curative.^[21]^ These alternatives include biologic therapy, targeted drug therapy, chemotherapy, and immunotherapy. Asymptomatic patients with a high tumor burden or slowly progressive PNETs may be treated with somatostatin analogs. Patients with highly differentiated PNETs or those progressing after somatostatin analog therapy can be treated with targeted therapies, which have shown promising results in terms of PFS. At present, sunitinib and everolimus have been approved for the treatment of PNECs.^[23]^ However, tumor regrowth following initial responses indicates that primary and acquired resistances may limit the efficacy of targeted therapies for PNEC. Systemic chemotherapy has limited efficacy, and overall survival (OS) is usually not >1 year.^[24]^ Clinical findings indicate that chemotherapy combined with immunotherapy can increase the objective response rate (ORR) to approximately 50%, but there are still many challenges in translating the benefits of ORR into improvements in OS and PFS.^[25]^

Although immunotherapy has demonstrated significant clinical efficacy in the treatment of several tumors, its application in PNECs remains in the early research stage. Some initial clinical studies suggest that NETs have some immune response. A phase II clinical trial evaluated the activity of ipilimumab combined with nivolumab in treating patients with microsatellite-stable advanced G3 NENs (mainly poorly differentiated tumors) achieving an ORR of 26% (95% CI: 11%–45%), a median PFS of 2.0 months, and a median OS of 8.7 months.^[26]^ Another study evaluated the efficacy of ipilimumab combined with nivolumab in patients with advanced NEN (90% were pretreated, 45% were high-grade NENs, and 7% had unknown primary origin). The ORR was 24%, and the disease control rate was 72%. The ORR for G3 and G2 patients with NEN were 31% and 23%, respectively.^[27]^ Another combination of ICIs, durvalumab plus tremelimumab, was evaluated in pretreated advanced NENs, resulting in an ORR of 9.1%, a 9-month OS rate of 36.1%, and a median OS of 5.9 months (95% CI: 2–9.7 months).^[28]^

Immunohistochemical analysis showed that the Ki-67 index was >20% in this case, indicating high proliferation activity. Combined with other examinations, the patient was diagnosed as advanced PNEC with multiple liver metastases. We preferred chemotherapy combinations as the first-line treatment, but the patient’s family refused to use chemotherapy drugs because of the side effects of chemotherapy. We considered that the patient had advanced liver metastatic cancer, and according to the data of ALTER G-001 cohort C, anlotinib combined with chemotherapy as the first-line treatment for unresectable liver metastatic gastrointestinal tumors, especially pancreatic cancer, responded well. In addition, according to the 2024 ASCO published penpulimab in combination with other drugs such as anlotinib, albumin-paclitaxel, and gemcitabine as the first-line treatment of advanced metastatic pancreatic cancer has good efficacy.^[29]^ Therefore, with the consent of the patient and his family, penpulimab combined with anlotinib capsules was used. In the course of treatment, the clinical symptoms were relieved by targeted therapy and immunotherapy alone. Laboratory and imaging examinations such as CT and ultrasound showed that the treatment effect was clear and the tumor remained stable, which was evaluated as SD.

Penpulimab is currently the only PD-1 monoclonal antibody in the world that uses a specialized IgG1 subtype structure. It eliminates antibody-dependent cellular cytotoxicity, antibody-dependent cellular phagocytosis, and complement-dependent cytotoxicity through Fc segment modification, making its structure more stable with fewer adverse reactions.^[30]^ In addition, compared with nivolumab and pembrolizumab, penpulimab exhibits a slower dissociation rate,^[31]^ resulting in more durable efficacy. Studies have explored the efficacy of penpulimab in combination with other drugs such as anlotinib, albumin-paclitaxel, and gemcitabine for the treatment of metastatic pancreatic cancer, in 66 evaluable patients, the ORR reached 50%, the disease control rate was 95.5%, and the median PFS was significantly extended to 8.8 months, with a median OS of 13.7 months.^[29]^ Penpulimab targets PD-1 to enhance the immune system’s attack on tumors, while anlotinib, a multitarget tyrosine kinase inhibitor, inhibits several kinases related to angiogenesis, thereby blocking the nutrient supply to tumors to inhibit growth and metastasis. Antiangiogenic drugs can alter the tumor microenvironment and work synergistically with immunotherapy to enhance efficacy.^[32]^ In addition, anlotinib has demonstrated effectiveness against pancreatic cancer in both monotherapy and combination therapy.^[33]^ In this case report, penpulimab demonstrated an enhanced therapeutic effect when combined with anlotinib. The patient’s PFS was 13 months, which was better than that reported in Sha et al’s^[29]^ study, indicating that penpulimab combined with anlotinib had certain clinical efficacy in treating PNEC, the patient achieved a longer survival time. Furthermore, grade 1 drug-induced liver injury and a slight increase in thyroid-stimulating hormone levels were observed during treatment, with no other serious adverse reactions. Compared with the efficacy and side effects of first-line treatments for PNEC, such as irinotecan/etoposide combined with cisplatin or temozolomide plus capecitabine, the treatment with penpulmab combined with anlotinib, in this case, is superior. Overall, the treatment was safe and effective.

As a case report, these findings cannot be generalized to broader PNEC populations, and interindividual variability may influence the universality of therapeutic responses. Due to clinical resource limitations, tumor genomic sequencing or microenvironmental analyses were not performed, precluding the identification of resistance mechanisms such as somatic mutations or tumor microenvironment heterogeneity, which restricts precision therapeutic optimization. In addition, the short postsecond-line follow-up duration hindered the evaluation of long-term OS and downstream treatment impacts. The absence of pancreatic primary lesion histopathology (due to family refusal of rebiopsy) further limited comprehensive molecular subtyping. Notably, minimal fluctuations in carcinoembryonic antigen and other biomarkers (CA: CA19-9, CA242, CA724, and CA50) during therapy underscored the limited reliability of single-marker monitoring, necessitating multimodal integration of imaging (CT/magnetic resonance imaging) and multiparameter biomarker profiling. Despite these constraints, this case provides novel insights for chemotherapy-sparing strategies for PD-L1 + PNEC. Future multicenter cohorts integrating molecular subtyping, extended follow-up, and predictive biomarker exploration are imperative to validate therapeutic feasibility and optimize clinical decision-making.

## 4. Conclusion

This article reports a case of a teenager with PNEC and multiple liver metastases who achieved disease stability after treatment with penpulmab combined with anlotinib; this response was maintained for 13 months. This case suggests that the combination of ICIs with antiangiogenic drugs may have some application prospects in the treatment of metastatic PNEC. Furthermore, this could potentially become a new first-line treatment option for patients who forgo chemotherapy.

## Acknowledgments

We are very grateful to the patients who provided cases.

## Author contributions

**Conceptualization:** Yafen Li.

**Writing – original draft:** Yafen Li, Jing Zhang.

**Investigation:** Jing Zhang, Yujie Liu.

**Writing – review & editing:** Jie Zheng, Zhiyu Ma.

**Data curation:** Yujie Liu.

**Funding acquisition:** Lu Zhao.

**Supervision:** Lu Zhao, Zhiyu Ma.
